# Malaria ecology along the Thailand–Myanmar border

**DOI:** 10.1186/s12936-015-0921-y

**Published:** 2015-10-05

**Authors:** Daniel M. Parker, Verena I. Carrara, Sasithon Pukrittayakamee, Rose McGready, François H. Nosten

**Affiliations:** Shoklo Malaria Research Unit, Mahidol-Oxford Tropical Medicine Research Unit, Faculty of Tropical Medicine, Mahidol University, Mae Sot, Tak Thailand; Faculty of Tropical Medicine, Mahidol University, Bangkok, Thailand; Nuffield Department of Medicine, Centre for Tropical Medicine, University of Oxford, Oxford, UK

**Keywords:** *Plasmodium*, Disease ecology, Thailand-Myanmar border, Biological diversity, *Anopheles*, Ethnic diversity, Anthropology, Demography, Malaria elimination

## Abstract

**Background:**

Malaria in Southeast Asia frequently clusters along international borders. For example, while most of Thailand is malaria free, the border region shared with Myanmar continues to have endemic malaria. This spatial pattern is the result of complex interactions between landscape, humans, mosquito vectors, and malaria parasites. An understanding of these complex ecological and socio-cultural interactions is important for designing and implementing malaria elimination efforts in the region. This article offers an ecological perspective on the malaria situation along the Thailand–Myanmar border.

**Discussion:**

This border region is long (2000 km), mountainous, and the environment ranges from thick forests to growing urban settlements and wet-rice fields. It is also a biologically diverse region. All five species of malaria known to naturally infect humans are present. At least three mosquito vector species complexes, with widely varying behavioural characteristics, exist in the area. The region is also a hub for ethnic diversity, being home to over ten different ethnolinguistic groups, several of which have been engaged in conflict with the Myanmar government now for over half a century. Given the biological and ethnic diversity, as well as the complex socio-political context, malaria control and elimination in the region is challenging.

**Conclusion:**

Despite these complexities, multipronged approaches including collaborations with multiple local organizations, quick access to diagnosis and treatment, prevention of mosquito bites, radical cure of parasites, and mass drug administration appear to be drastically decreasing *Plasmodium falciparum* infections. Such approaches remain crucial as the region moves toward elimination of *P. falciparum* and potentially *Plasmodium vivax*.

## Background

Malaria is one of the most important diseases in the world, with an estimated 198 million cases and 584,000 malaria-related deaths in 2013 [1]. In the last decade, there has been much progress in malaria control and global numbers of symptomatic cases and mortality have been decreasing. This pattern is evident in Southeast Asia, where the spatial distribution of malaria is heterogeneous, with some regions having little or no malaria and others remaining endemic, with seasonal cases. In Southeast Asia cases often cluster along international borders [2–4]. The Thailand-Myanmar border is one such example, being a point of convergence for different nations, with differing economic development, public health infrastructure and policy, and sociocultural and political situations.

In Thailand, for example, concerted efforts at malaria elimination began in the mid-1960s and while these efforts shifted toward malaria control in the 1970s, elimination from much of the central provinces was quite effective. For the last several decades, most of central Thailand has been malaria free. However, international borders, especially with Cambodia and Myanmar, remain a refuge for both *Plasmodium vivax* and *Plasmodium falciparum* parasites, despite vast improvements in the overall malaria situation even within these sub-regions.

The situation in Myanmar is less well-known. With over half a century of political turmoil and conflict, public health infrastructure remains underdeveloped. Central plains regions continue to have malaria, however these regions are also most likely to benefit from the few public health resources that are available. Border regions are the homelands and territories of ethnic militias and rebel groups and are largely outside the purview of government-based health services. These regions are therefore likely to have the worst public health situations, but because there is also a dearth of reliable and relevant public health data, a comprehensive understanding of the public health scenario here is lacking. Large amounts of human migration across and along the border region likely contributes to the complex malaria situation, with migrants potentially transporting malaria parasites or exposing themselves to epidemiological landscapes with which they are unaccustomed [5, 6]. For both Myanmar and Thailand, this border region represents the frays of society, a convergence zone where the social and physical landscape, the biological and cultural diversity, and the collision of different national programmes and interests create what from the outside (at least) can appear to be an impossible problem to tackle.

This paper presents an ecological approach to understanding the current malaria situation in this border region. An ecological understanding includes the landscape, the parasite and the vector biological diversity, the human host ethnic diversity, and the demographic attributes that characterize this region and contribute to the current public health situation. The paper concludes that despite these complexities, real public health improvements have been made and the future seems to be one in which these public health successes will continue.

## Landscape

**Fig. 1:**
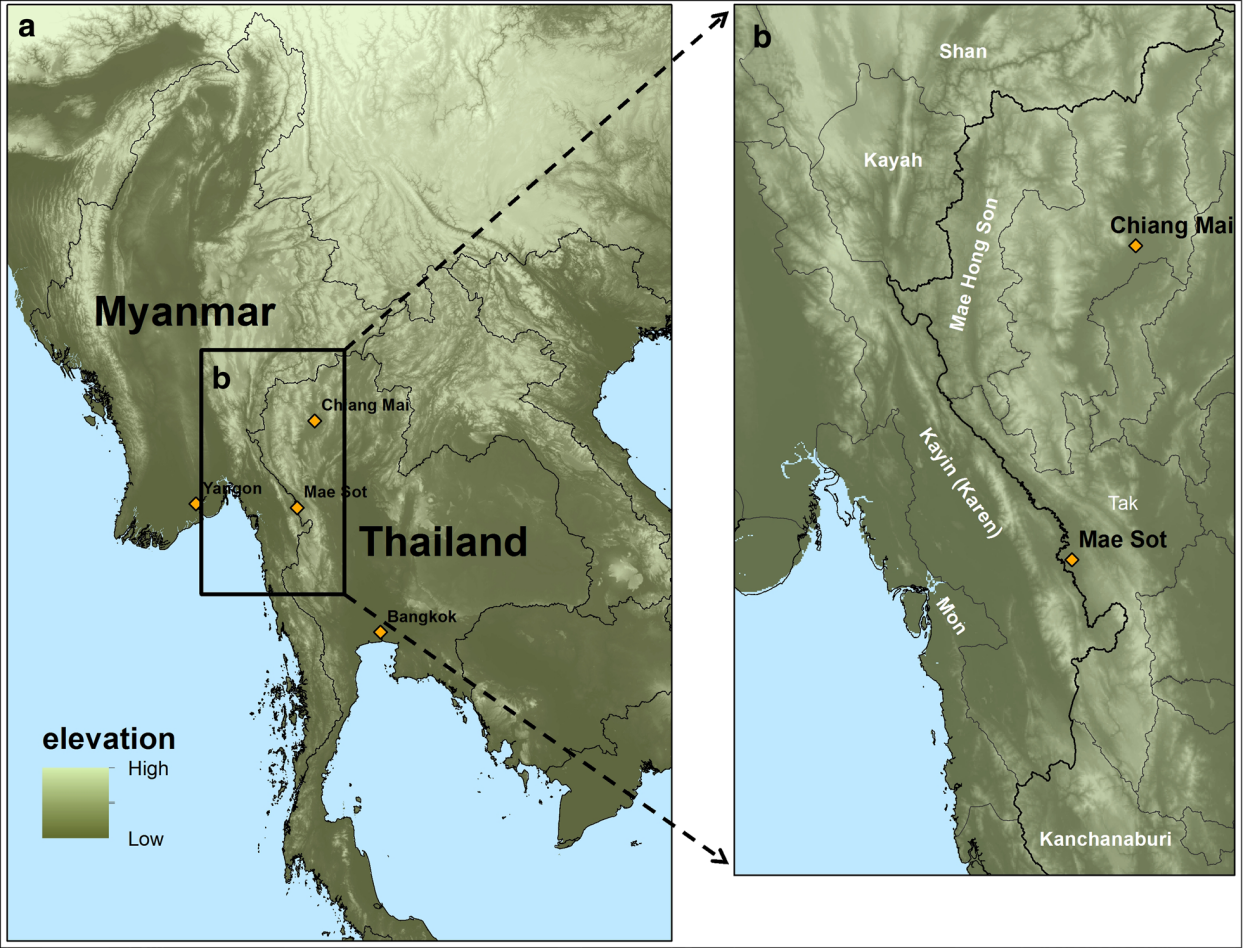
**a** Elevation map of mainland Southeast Asia. **b** Map of Thailand–Myanmar border area, including border states of Myanmar and provinces of Thailand. Map created using ArcMap 10.2

The international border between Thailand and Myanmar stretches almost 2000 km. The population centres of both nations lie in their central plains regions, which are split along the international border by a natural buffer zone formed by the southern reaches of the Himalayas (Fig. 1). This landscape includes watersheds, river basins and valleys which are filled with primary and (mostly) secondary or tertiary forests, agricultural fields, plantations, and occasionally dense pockets of human settlements ranging from refugee camps, agricultural villages, to river and border trading towns.

Much of the area is difficult to traverse and has therefore been slow to urbanize in comparison to other regions, such as the heavily populated lowlands near Yangon and Bangkok. While the area has undoubtedly been affected by human environmental degradation, it remains a biologically diverse region, related to the diversity of ecological habitats and relatively small human population density.

The implications of these geological and environmental aspects are multifold. With regard to malaria ecology, the environment and landscape has ecological and behavioural implications for humans, mosquitoes and parasites. All five species of malaria known to naturally infect humans exist in these borderlands, with *P. falciparum* and *P. vivax* dominating; at least three major mosquito vector complexes occur in the same area; and well over ten different ethnic groups call the region home [2, 7]. The ecological, sociocultural, and linguistic profile of the region is one of diversity (biological and ethnic) as well as complexity.

Furthermore, climate change and deforestation may impact each of the three organisms in the malaria system, although the precise outcomes are difficult to predict. Increased average temperatures may alter preferential mosquito vector habitats. Deforestation can reduce preferential habitats for some mosquito vectors while simultaneously increasing it for others [8]. Both these factors are related to (a result and likely driver of) changes in human behaviour, settlements and ecology.

## Malaria parasite populations

In 2014, 21 % of all confirmed malaria cases within Southeast Asia were in Myanmar. Within Myanmar, an estimated 74 % of all cases were attributed to *P. falciparum* whereas within Thailand around 44 % were attributed to *P. falciparum* [1]. *Plasmodium ovale*, *Plasmodium malariae* and *Plasmodium knowlesi* infections are all found along the border, but are rare [9, 10].

Throughout the border region, the contribution of *P. vivax* to overall malaria morbidity is increasing. However, vivax epidemiology differs from falciparum epidemiology in that most vivax cases are the result of relapse (reemergence of parasites in the blood from hypnozoites) rather than novel infections [11–13]. Treatment of *P. vivax* is complicated by hypnozoites because hypnozoitocidals can also have adverse effects in humans with glucose-6-phosphate dehydrogenase deficiency (G6PDd), a human enzyme disorder that is widespread in Southeast Asian populations [14, 15]. Testing for G6PDd has until recently been possible only in laboratory settings.

Drug resistance in local parasite populations is also a major concern. *P. falciparum* parasite populations in the region have an apparent proclivity for developing resistance to anti-malarials, having historically been among the first to develop resistance to successive lines of drugs [16, 17]. Resistance to chloroquine arose in this region (and South America) in the late 1950s and the Southeast Asian strain spread to Africa within a few decades [18]. By the end of the 1970s the combination therapy sulfadoxine and pyrimethamine (SP) was also ineffective. Treatment was then largely focused on mefloquine but by the late 1980s resistance to this drug was also apparent. More recently, resistance to artemisinin and its derivatives (the last widely effective anti-malarial) appears to have emerged in Cambodia (near the Thailand-Cambodian border) and along the Thailand-Myanmar border [19–21].

Several hypotheses (not mutually exclusive) have been proposed to explain this pattern, broadly related to pharmacokinetics, transmission levels and population genetics. One has to do with exposure of parasites to sub-therapeutic levels of anti-malarials, through the misuse of medications, the circulation of sub-standard anti-malarials [22] or through mass administration of sub-therapeutic anti-malarials (e.g., chloroquine in salt) [23]. A sub-lethal concentration of anti-malarial in the blood may act as a low-level pressure through which the proportion of mildly resistant strains arise within the infected host. Anti-malarials with long half-lives create an environment through which host parasite populations have lengthy exposure and relatively longer periods during which de novo resistance mutations can arise and subsequently increase in number. If these parasites are subsequently capable of surviving and spreading, and if strong anti-malarial resistance is acquired in a step-wise pattern (where low-level resistance can lead to high-level resistance) then anti-malarial resistance may emerge. Presumably, exposure to low levels of anti-malarials is not unique to Southeast Asia.

One proposed explanation for the persistence of drug resistance in the region is related to transmission intensity (number of infectious bites per person per unit time) and multiplicity of infections (carrying multiple strains of a single parasite species at the same time). Compared to places like sub-Saharan Africa, transmission in Southeast Asia is generally considered to be low [24]. Since infected persons in high transmission areas are more likely to have been bitten by multiple infectious mosquitoes, the multiplicity of infection should also be higher in high transmission areas [25, 26]. A mosquito taking a blood meal from an infectious person in a high transmission area should therefore be more likely to take multiple strains [25, 26], and since genetic recombination occurs within the mosquito, genetic mixing can occur. In multigenic drug-resistant strains, resistance can then be lost across generations through recombination. Conversely, in low transmission areas, once a drug resistant form of parasite has emerged it may persist for longer periods of time. While this hypothesis isn’t directly related to the emergence of novel resistance genes, it could help explain the persistence of multigenic resistance in low-transmission settings.

It has also been suggested that infected persons in low-transmission settings are less likely to have acquired immunity to malaria parasites over their lifetimes, and are therefore likely to experience higher parasite loads during infections. Patients who have higher parasite loads during infections are also likely to seek treatment. If sub-therapeutic levels of anti-malarials are present in the infected host, and if a higher biomass of parasites is exposed, then there are more opportunities for selection of resistant or partially resistant parasites [23, 27].

The concept of transmission level in the region is, however, problematic from a spatial perspective. Even in low transmission regions there are small pockets of high transmission, and transmission can vary seasonally [24, 28, 29]. When transmission statistics are aggregated at subnational, national, or regional units this heterogeneity is lost. The related hypothesis regarding acquired immunity may also be flawed given several recent findings of large proportions of asymptomatic falciparum malaria in regions considered to have low transmission [30–32]. Perhaps some people in low transmission areas are exposed to enough malaria to have acquired immunity to malaria, or perhaps acquired immunity can occur even in the absence of frequent reinfection [33].

A further hypothesis has been that parasite strains from Southeast Asia exhibit a ‘hypermutator’ phenotype. For example, some in vitro evidence that *P. falciparum* parasites from Southeast Asia develop resistance mutations faster than West African counterparts [34]. However, this hypermutator behaviour was shown in laboratory strains that have existed under laboratory conditions for many generations. Brown et al. took field strains with associated clearance phenotype data and looked for differences in substitution rates across parasites populations from Southeast Asia (Bangladesh, Cambodia, Laos, Myanmar, Thailand, Vietnam) and West Africa (Mali), and found no evidence of increased mutation rates in Southeast Asian parasite populations [35]. However, parasites from Mali did exhibit much shorter blocks of linkage disequilibrium in comparison to parasites from Southeast Asia, which is expected in settings with more mixed strain infections and subsequently more genetic mixing through recombination.

Most literature and research regarding drug resistance in malaria has focused on *P. falciparum*, however antimalarial resistance in *P. vivax* (especially with regard to chloroquine) is a growing concern [36–39]. Chloroquine resistant vivax (CRV) has been reported in Southeast Asia now for several decades, in Myanmar since the early 1990s [36] and more recently in Thailand [38]. CRV appears to be widespread throughout vivax endemic regions [39]. *P. vivax* is increasingly the largest contributor to overall malaria morbidity along the Thailand-Myanmar border and, as such, drug resistant vivax malaria may increasingly be a major cause of concern.

Regardless of the reason for the repeated emergence, persistence and spread of drug resistance within and beyond this region, the existence and continued emergence of multidrug-resistant malaria here is a complicating reality that requires thoughtful and bold approaches.

## Mosquito populations

The most important mosquito vectors in this region include *Anopheles dirus* sensu lato, *Anopheles minimuss.l.* and *Anopheles maculatuss.l.* Further work (both in the field and laboratory) may reveal other important vector species in the region [40, 41]. The relative importance of mosquito vectors is directly related to their human blood-feeding behaviour, which is in turn influenced by several factors, including host preference, the availability of hosts and contact patterns between vectors and hosts [42]. Indoor- versus outdoor-feeding behaviours (endophagy and exophagy, respectively) are also important aspects of feeding behaviour in mosquito vectors and ultimately in the ecology and epidemiology of malaria.

### *Anopheles dirus* sensu lato

*Anopheles diruss.l*. is made up of at least seven species: *Anopheles dirus* sensu stricto, *Anopheles crascens*, *Anopheles scanloni*, *Anopheles baimaii*, *Anopheles elegans*, *Anopheles nemophilous*, and *Anopheles takasagoensis* [43]. The Thailand-Myanmar border area marks a suture zone between *An. baimaii* and *An. dirus* [43].

*Anopheles diruss.l.* is almost always associated with deep forests, foothills and primary larval habitats are typically in temporary standing or slow moving water under shade [43, 44]. During the rainy season, they may expand their habitat to forest edges, putting them closer to human settlements [43, 45]. They can also adapt to the edges of clearings [44–46], to plantations [47], and have even been found in rice fields [48]. *Anopheles diruss.l.* are relatively long-lived and tend to be highly anthropophilic (though see [49]), making them a highly effective vector. While they are typically considered exophilic, biting can occur as frequently indoors as it does outdoors in open houses in forests [43, 47, 50, 51]. Feeding patterns (early *versus* late) are sometimes contradictory, even in the same site and species across different years or locations [28, 43].

### *Anopheles minimus* sensu lato

The *Anopheles minimus* complex [*An. minimuss.l.*) is made up of at least three sibling species (*An. minimuss.s.* (previously *An. minimus* A), *Anopheles harrisoni* (previously *An. minimus* C) and *Anopheles yaeyamaensis* (previously *An. minimus* C)]. *Anopheles minimuss.s*. and *An. harrisoni* are both present in Southeast Asia (*An. yaeyamaensis* is confined to the Ryukyu archipelago in Japan) [52]. Historically, there has been confusion with regard to *An. minimuss.l.* phylogeny, partially because of the difficulty in morphologically distinguishing between species and because of its behavioural plasticity (diverse and changing behaviour patterns). Several behavioural studies have failed to differentiate between *An. minimuss.s.* and *An. harrisoni* and both species are probably highly opportunistic [52].

Larval habitats usually include small streams or canals, with clear, clean and cool water, in partial shade [52–54]. *Anopheles minimuss.s.* occupies a wide range of habitats, including both dense forests and open agricultural fields—it appears particularly well suited for rice paddy agricultural systems in forested and hilly regions. Conversely, *An. harrisoni* habitats are linked to recently altered landscapes (deforested agricultural fields, etc.) [52, 55, 56].

Several studies have indicated that the presence of cattle can influence host choice behaviour in both *An. minimuss.s*. and *An. harrisoni* [42, 57]. *Anopheles minimuss.s.* appears to more frequently feed on humans in areas where cattle are not present. *Anopheles harrisoni* is generally zoophilic, but more studies are needed to better understand this species. *Anopheles minimus* s.s. will clearly change its behaviour when exposed to different environmental stimuli, meaning that behavioural characteristics are not confined to species. In Thailand, *An. minimuss.s.* is considered endophagic while *An. harrisoni* is typically exophagic [42, 58].

*Anopheles minimuss.s*. tends to be a late biter (after 22:00) while biting times for *An. harrisoni* in Thailand appear to peak twice, once in the early evening (18:00–20:00) and again in the early morning (24:00–02:00 or 03:0006:00) [42, 52, 58–60]. Bed net usefulness may therefore be limited to *An. minimuss.s*. [42].

### *Anophelese maculatus* sensu lato

The *Anopheles maculatus* complex (*An. maculatuss.l*.) has recently been reclassified as a super complex, which is in turn composed of several sub-groups (the Maculatus group and the Maculatus and Sawadwongporni sub-groups) [61–63]. It is made up of nine sibling species: *Anopheles dispar*, *Anopheles greeni*, *Anopheles pseudowillmori*, *Anopheles willmori* in the Maculatus group, *An. maculatuss.s.* and *Anopheles dravidicus* in the Maculatus sub-group, *Anopheles notanandai*, *Anopheles rampae*, *Anopheles sawadwongporni* in the Sawadwongporni sub-group [61–63].

*Anopheles maculatuss.l.* is typically found in or near hilly, forested regions. It is also known to inhabit forest camps (logging camps) and mountain areas above 1200 m [64]. It breeds in shallow, rocky and sandy pools near clean rivers or streams, with direct sunlight. The larvae have been found together (e.g., in Tanintharyi Division, Myanmar) with *An. minimus* larvae [65].

Seven species of the *An. maculatus* group have been identified in Thailand, though not all are malaria vectors. *Anopheles maculatuss.s*., *An. pseudowillmori* and *An. sawadwongporni* have been implicated as malaria vectors in Thailand. *Anopheles maculatuss.l*. has also been considered an important vector in the southern reaches of the Thailand-Myanmar border region (Tanintharyi Division of Myanmar). Elsewhere in Myanmar it is considered a secondary vector. Several studies have indicated that it reaches peak population densities during the cold, dry season (January) in Myanmar, making it potentially important as a carry-over vector for malaria parasites during low incidence times of the year. It is known to opportunistically feed on both animals and humans and mainly during the first and second quarters of night (18:00–24:00) and typically exhibits exophagic behaviour.

## Vector ecology and control strategies

The biological and behavioural diversity of mosquito vectors within the border region complicate the effective use of preventive methods. For example, insecticide treated nets (ITNs) alone are unlikely to be sufficient because of residual malaria transmission (transmission that persists after effective vector control using ITNs or indoor residual spraying in situations where the vector population is susceptible to the insecticides being used and where coverage is ensured). Residual transmission is then related to early or outdoor biting behavior in mosquito vectors and human behavioral factors (e.g. occupational exposures to various ecological settings).

Previous studies (now over 20 years old) in the region on bed net efficacy have been relatively contradictory. One study in school children indicated high compliance in ITN usage and a 38 % reduction in parasitemic falciparum infections [66]. Another study took place in three different refugee camps and looked at potentially protective effects of different bed net types (non-treated single person (NIB), permethrin-impregnated single person (PIB), and family size non-impregnated (FNIB)) in pregnant women [67]. This study showed that non-treated bed nets had no protective effect with regard to malaria and general anemia. In one camp, pregnant women who used either FNIB or PIB had less malaria, though the effect size was small (RR: 1.67; CI 1.07–2.61). There was no difference by bed net type and malaria incidence in the other two camps. However, pregnant women who used FNIB and PIB in any of the camps had significantly lower incidence of anemia. Presumably these anemia cases were related to subpatent malaria. Ultimately, ITNs only work when they are in use, which typically occurs in very specific spaces and times (i.e. in homes, at night) and can miss high-risk subgroups of a population (people who work outdoors, at night, etc.)

Insecticide spraying is also unlikely to have far reaching effects since it will be confined to specific places and times and because of the growth and spread of insecticide resistance, which is known to exist in Southeast Asia, but has not been well studied along the Thailand-Myanmar border [68]. A few studies have indicated that while spraying can reduce vector species population sizes, these decreases do not halt transmission because of the diversity of vector species and their preferred habitats [47]. Also of concern with vector focused efforts are behavioural plasticity (some mosquito vectors alter their targeted blood feeding habits) and behavioural evolution (selecting against some feeding times and preferences may lead to shifts in the feeding times and preferences within a single species) [42, 47, 69].

Other vector-focused approaches include personal protective clothing (either treated or not), the application of insect repellants via sprays or topical ointments, spatial repellants (long-lasting repellant emanators), and diversion through traps or bait [70]. Personal use repellants have shown effectiveness at reducing exposure to mosquito bites [40, 70], but have not generally shown a reduction in the risk of malaria infection [71, 72]. Spatial repellants, traps and baits may offer further options but their effectiveness has not yet been validated [73].

The diversity vector species and behaviours (even within the same species), combined with the mobile nature and subsistence strategies of many of the human populations in this region, confound vector focused strategies. Approaches that combine multiple strategies (ITNs in combination with repellants and perhaps baits or traps) are likely to be the only viable vector focused solution. Furthermore, such efforts must be used in combination with parasite and human focused approaches.

## Human populations

The international border between Thailand and Myanmar is home to people of Thai, Burmese, Chinese, Indian, Laotian, Vietnamese, and Bangladeshi descent, living in clusters, mainly in major border trading towns. These groups could be further sub-divided into minority groups, including religious minorities. The bulk of the population is made up of a collection of different minority groups collectively referred to as ‘hill tribes’. While there are numerous hill tribes throughout mainland Southeast Asia, within this border region are Karen, Hmong, Lisu, Mien, Lahu, Meo, Padaung, and Kayah/Karenni peoples. Other major ethnic minority groups along the border include the Shan and the Mon, in the northern and southern portions of the border, respectively.

Major population centres occur near rivers which have historically been the most reliable sources of year-round movement and transportation. However, most people along this border region live in rural areas, with some inhabiting extremely remote, difficult-to-access locales along both sides of the international border. Also, on both sides of the border, new roads are being built, old roads are being improved so that they may be used year-round, and many sub-regions are undergoing economic development. While such development is always unevenly distributed, it will almost certainly lead to changes in economy, demography and rural–urban inhabitation and movement patterns. Further still, a series of refugee and internally displaced person (IDP) camps dot the landscape [74, 75]. A few of these camps rival the largest towns in this region with regard to population size and density (especially Mae La camp in Tha Song Yang District, Tak Province), as well as ethnic diversity (including refugees even from westernmost regions of Myanmar) [74, 75].

With regard to malaria epidemiology and ecology, the rise and development of urban centres seems to have disrupted malaria cycles. The major towns and cities along the international border currently have very little or no malaria aside from cases that are likely to have been obtained outside of the towns. The reasons for this pattern are multifold.

**Fig. 2:**
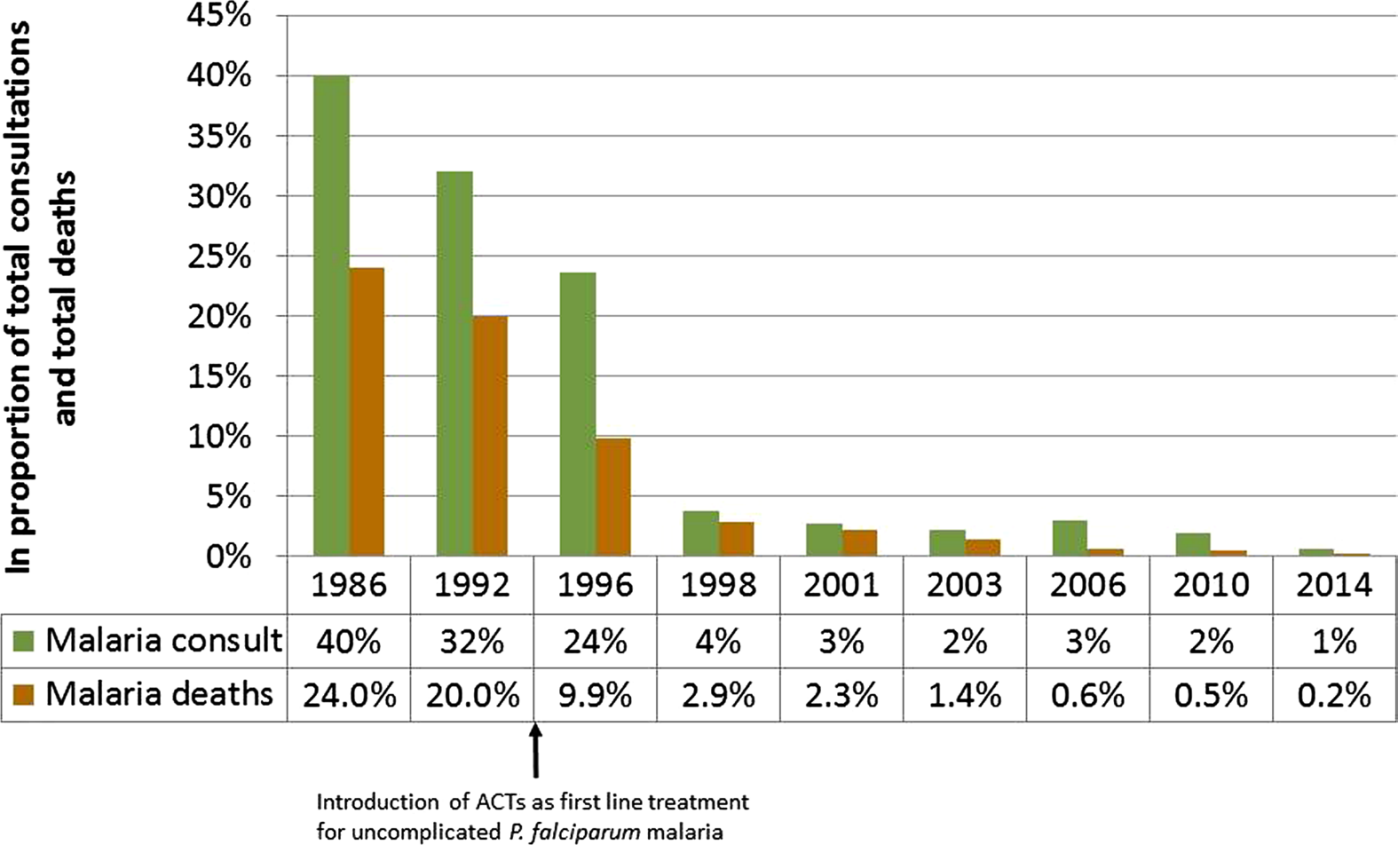
Decreasing burden of *Plasmodium falciparum* malaria over time in the nine refugee camps along the Thailand–Myanmar border. Data are not available for each year, resulting in gaps in the timeline. After the introduction of artemisinin-based combination therapy (ACT) in 1995–1996 there was a drastic reduction in the burden of malaria [124]

Access to early diagnosis and treatment of malaria can have a profound impact on overall incidence (Fig. 2) [76, 77], and health care providers tend to cluster in urban centres. Public health and sanitation works are more likely to be in place and to have sufficient funding to be maintained in urban areas.

Environmental changes are also related to decreases in malaria cases. The larval stages of several malaria vector species thrive in clean water sources and once water sources have become polluted, their population numbers decrease. Concrete, pavement and other changes to the environment can also decrease preferable habitats for the most important malaria vectors. Concrete also reduces habitat that is favourable to livestock. Given that some species may be drawn to livestock, and can arbitrarily feed on proximate humans, this can lead to a reduction in attractive feeding zones for mosquitoes.

Within the border region, hill tribe members tend to be at greatest risk of infection, and *P. falciparum* infections cluster in adult males [77, 78]. This pattern is the result of multiple factors, largely centred on environment and exposure. Many hill tribe persons seek their livelihoods through subsistence or commercial farming. Others make a living as hired labourers, frequently in the agricultural sector. Such people are tied, at least seasonally, to the land [79–82]. As with other agricultural populations, the implication is that there are pulse-like population dynamics, including births, migrations and population densities that revolve around changes in the agricultural calendar and differ slightly by ecotope [83–87]. These population dynamics can influence malaria ecology in several ways, chiefly by introducing susceptible persons to different ecological zones, potentially exposing them to different malaria vectors, at different times of the year [88].

Several of the ethnic groups along the Thailand-Myanmar border have also been engaged, over the last half century, in off-and-on warfare with the Myanmar military [89, 90]. Soldiers traverse through and sleep in heavily forested areas, frequently travel long distances, and may not have adequate materials (tents and mosquito nets) for preventing mosquito bites [91]. Their communities may also be at increased risk of infection if soldiers transport and introduce these parasites to their home villages.

This long-standing warfare has had enormous implications for non-military members of the population [89, 92]. At times, individuals, families, villages and even collections of villages have been forced to relocate as a result of fighting [89, 93]. Sometimes the relocations occurred across the border, with thousands of Karen, Shan and other ethnic minority groups moving to relative safety on the Thai side of the border [74]. Others have relocated near the border in IDP (internally displaced person) camps. Some of these camps have become semi-permanent settings, now in existence for up to 30 years. Other refugees have occasionally spent time in makeshift villages and camps until they are able to either move back to their home villages in Myanmar or are able to move into the more permanent refugee camps.

For those who have found their way to the more permanent camps, overall health has improved in many ways. Whereas malaria was one of the largest contributors to overall morbidity and mortality 30 years ago [78, 94], today it is one of the smaller contributors (Fig. 2) [76]. This is largely the result of focused work by governmental and non-governmental organizations that have devoted their efforts to these vulnerable populations. However, for those who have been unable to move into these well-established camps, the health care situation can be lacking or even non-existent. Those who make a living on the Thai side of the border do have health care options, however many ethnic minority people have no nationality, speak very little or no Thai, and are unable to easily travel to health care centres [95]. Since many live in conditions of extreme poverty they may not have adequate housing or protective mosquito nets to cover all family members, may not have the knowledge necessary to help prevent malaria infections, and may be more prone to participating in subsistence behaviours that put them at higher risk of malaria infection [96].

To add to this complexity, the ability of such long-term camps to remain in Thailand has never been secure. Thailand has never signed to the 1951 Refugee Convention or the 1967 Protocol Relating to the Status of Refugees, and refugee camps *per se*, are not technically allowed to exist on the Thai side of the border [97]. The existence of these long-term camps is questionable, with frequent rumours and political movement towards repatriation of camp inhabitants to the Myanmar side of the border. Those fleeing conflict in Myanmar do not have the normal refugee status in Thailand, but are allowed to remain temporarily in the camps, which may or may not be allowed in the foreseeable future.

## Human population movement

Human population movement has frequently been cited as a major factor in the persistence of malaria along this international border [3, 4, 16, 98–102] and there are several ways that migration can influence malaria epidemiology [6, 103, 104]. Migrants from a malaria-endemic region can bring the parasite with them when they move to a new, non-malarious region [105]. If that region has capable vectors, the disease could take hold in the new region. Likewise, migrants in non-malarious regions may pick the parasite up when they visit malaria-endemic areas and bring it back to their place of origin. A similar scenario can explain the spread of drug-resistant strains [16, 27, 106, 107]. Migrants who visit areas where drug-resistant strains exist can move those strains to new places, regardless of whether or not the new place already has malaria (assuming a competent vector species is present). The transportation of malaria parasites is not the only concern. Individuals who move into malarious regions also increase the number of susceptible individuals in those regions, potentially influencing epidemiological dynamics. If they come from a non-malarious region, they may not have acquired immunity, meaning that morbidity and even mortality can be increased [108, 109].

Much of the regional migration within the Greater Mekong Sub-region of Southeast Asia is centred around Thailand. Thailand is wealthier than many of its neighbouring, landlocked countries. For example, the per capital GNI in 2012 ($5210) is roughly six times higher than that of Cambodia ($880), four times higher than Laos ($1260), and five times higher than Myanmar ($1035) [110]. It therefore acts as an economic magnet or ‘pull’ factor for migrants who are seeking jobs and better wages [111, 112].

Most of the international migration into Thailand comes from Myanmar and several researchers have claimed that this cross-border movement is a major factor in malaria morbidity and mortality in Thailand [3, 113, 114]. Some studies have indicated a higher prevalence of malaria in migrants (within Thailand) who are from Myanmar [113, 114], leading to the suggestion that migrants (especially from Myanmar) are a major public health problem for Thailand. On the other hand, Thailand has a policy of providing free diagnosis and anti-malarials to patients with confirmed malaria, regardless of their nationality. This leads many people from the Myanmar side of the border to cross solely for the chance to receive anti-malarial treatment. Most health care providers are located in major border towns and cities, along major roads and rivers, and people from the Myanmar side flock to such services. Such ‘migrants’ are recorded in the epidemiological data, leading to a situation in which malaria case numbers in foreigners on Thai soil are high but not necessarily because being a foreigner carries an inherent risk for malaria infection (see [115, 116] for a similar pattern with HIV/AIDS patients). However, such migrants can import malaria parasites if mosquitoes feed on them after they have arrived on Thai soil.

While there have been many malaria studies in this region, a detailed understanding of the relative importance of human movement patterns versus occupational and behavioural risk factors is lacking, with regional armed conflict creating a significant barrier. Directly linking human movement patterns to the risk of malaria infection has also been extremely difficult. However, the potential influence of human movement and migration patterns on malaria ecology and epidemiology remains an important and worthwhile topic for continued and new investigations.

## Conclusions

The border region connecting Thailand and Myanmar is a mixing place, a point of entropy, for many different interconnected factors. It is a regional hot spot for biological diversity, and that diversity extends into the realm of medical importance through many extant and indigenous parasites and mosquito vectors. It is also a point of increased ethnic diversity, with majority and minority groups coming together historically at a point between two ancient kingdoms, in unique ecological zones that are well-suited for various subsistence practices, as well as for relatively high-volume trade and subsequent economic opportunities. The spatial demography of the region is diverse, ranging from population-dense border towns and refugee camps to evenly distributed farming villages in lowland rice paddy settings and highland farms. As the joining point of two major nations, and a refuge of several rebel military groups, the region is heavily militarized. It is a meeting place for two very different health care systems—that of Myanmar and Thailand—with little overlap, historical cooperation, or agreement on medical protocol or strategy. The result is a confusing and complex convergence zone for both mosquito vector biological diversity and human cultural diversity along the Thailand-Myanmar border [117, 118].

Despite this complex and difficult-to-understand scenario, the malaria situation has greatly improved in the region over the last several decades [77]. On the Thai side of the border falciparum malaria is almost completely absent and vivax malaria remains in small numbers and limited settings only. On the Myanmar side, current efforts are underway to drastically reduce an already dwindling malaria burden. These efforts are, however, threatened by the existence of strains of increasingly drug-resistant falciparum malaria [119]. Yet through increased attention by global funding agencies, as well as increased cooperation between governmental, non-governmental organizations and academic institutes, it may be possible to reduce falciparum populations to such an extent that such resistant strains are halted before they become major public health disasters.

A series of elimination projects, currently aimed directly at falciparum but likely to expand to vivax malaria too, are already in progress along the Thailand-Myanmar border. It is a multipronged approach which includes creating easier access to quick diagnosis and treatment, cross border work, prevention efforts, radical cure of malaria infection, and in some cases mass drug administration [120]. Malaria persists in remote regions at least partially because of a lack of access to quick diagnosis and treatment and there is a widespread effort to create and maintain village based malaria posts (MPs) to address this issue. MPs are staffed by local villagers who have been trained to diagnose malaria using rapid diagnostic tests and to subsequently provide anti-malarials for malaria positive cases. Such efforts have drastically reduced falciparum infections throughout much of the region [121]. As a result of both geographic and political constraints, some regions remain easiest to approach for health related purposes through cross border work (i.e. accessing border communities on the Myanmar side via the Thailand side) [122]. Prevention efforts are also under way, including community-based education about prevention, insecticide treated bed nets and protective clothing (treated with insect repellant) and topical repellants. For both falciparum and vivax infections, radical cure is crucial to halting transmission. Currently in remote settings this is only feasible for falciparum infections as the radical cure is unlikely to result in adverse symptoms in patients. Radical cure for vivax infections can be dangerous for some individuals with G6PDd [11, 123]. Current field validation tests are underway for rapid and accurate G6PDd diagnostics so that radical cure of vivax infections can quickly and safely be employed throughout the region. Finally, in cases where submicroscopic and asymptomatic cases are especially prevalent, it may be necessary to undergo mass drug administrations to eliminate the reservoir. Such efforts for falciparum malaria are already under way [120].

Early efforts suggest that cooperation among many different actors, with diverse visions, wants and hopes, is possible despite the complications and inherent difficulties. Successful reduction and even elimination of falciparum and vivax malaria through such consortia would be much more than a major public health success story.
